# Adrenal Cushing’s Syndrome Treated With Preoperative Osilodrostat and Adrenalectomy

**DOI:** 10.1016/j.aace.2022.10.001

**Published:** 2022-10-10

**Authors:** Risha B. Malik, Anat Ben-Shlomo

**Affiliations:** 1 Division of Endocrinology, Diabetes & Metabolism, Cedars-Sinai Medical Center, Los Angeles, California; 2 Multidisciplinary Adrenal Program, Department of Medicine, Cedars-Sinai Medical Center, Los Angeles, California

**Keywords:** Cushing’s syndrome, osilodrostat, adrenalectomy, adrenal insufficiency, ACS, adrenal Cushing's syndrome, ACTH, adrenocorticotropic hormone, BID, twice daily, DST, dexamethasone suppression test, mUFC, mean urinary free cortisol, NET, neuroendocrine tumors

## Abstract

**Background/Objective:**

Reducing severity of Cushing’s syndrome caused by an adrenal adenoma (adrenal Cushing’s syndrome [ACS]) might decrease morbidity and mortality risk in adrenalectomy. We used off-label osilodrostat, approved in the United States for pituitary Cushing’s disease, to reduce cortisol levels and disease severity before adrenalectomy 3 weeks later.

**Case Report:**

A 48-year-old woman with a 6-year history of obesity, depression, and anxiety and 3-year history of diabetes and hypertension was admitted with vomiting and lumbar back pain. Facial plethora and hirsutism, posterior cervicothoracic fat pad, and truncal obesity coupled with morning serum cortisol >13 μg/dL after 1 mg oral dexamethasone suppression, urinary free cortisol 1324 μg/24hr (4.0-50.0 μg/24 h), and adrenocorticotropin <5 pg/mL (6-50 pg/mL) confirmed ACS. Computed tomography with contrast revealed a 3.4-cm right adrenal mass. Osilodrostat 2 mg twice daily initiated at discharge was increased to 4 mg twice daily on day 6. Three days later, she reported nausea, vomiting, and fatigue. Despite 7.2 μg/dL morning cortisol, adrenal insufficiency was suspected; osilodrostat was reduced to 2 mg twice daily and maintenance oral hydrocortisone 20 mg daily was added with symptom resolution. Prior to adrenalectomy, morning cortisol was 5.1 μg/dL, fasting glucose was 122 mg/dL, and she self-discontinued diabetes medications. Hypertension remained unchanged (149/100 vs 151/94 mmHg). Adrenalectomy revealed a 3.4-cm focally pigmented adrenocortical adenoma.

**Discussion:**

Three-week treatment of overt ACS with off-label osilodrostat reduced cortisol and glucose levels before curative adrenalectomy. Abrupt cortisol reduction led to suspected adrenal insufficiency managed with maintenance hydrocortisone.

**Conclusion:**

Osilodrostat might help reduce ACS severity before adrenalectomy. Adrenal insufficiency is a risk but can be safely managed with hydrocortisone.

**Highlights**

• Osilodrostat inhibits CYP11B1, a steroidogenic enzyme metabolizing 11-deoxycortisol to cortisol in the adrenal cortex.
• Osilodrostat is approved in the United States to treat Cushing’s disease caused by an adrenocorticotrophic hormone (ACTH)-producing pituitary adenoma in patients for whom pituitary surgery is not curative or is not an option.
• Off-label, short-term use of preoperative osilodrostat in a patient with Cushing’s syndrome due to an adrenal cortisol-secreting adenoma decreased elevated cortisol and glucose levels prior to adrenalectomy.
• Adrenal insufficiency is common with rapid up-titration of osilodrostat and requires treatment with concurrent hydrocortisone.

**Clinical Relevance**

The steroidogenesis inhibitor osilodrostat is approved in the United States to treat Cushing’s disease caused by a pituitary adenoma. We used osilodrostat to reduce cortisol and glucose prior to adrenalectomy in a patient with Cushing’s syndrome caused by an adrenal adenoma. Adrenal insufficiency upon up-titration was successfully treated with hydrocortisone.

## Introduction

Adrenocorticotropin (ACTH)-independent adrenal Cushing’s syndrome (ACS) is caused by autonomous secretion of cortisol from a benign adrenal adenoma or carcinoma. The incidence of ACS was recently calculated at 91 per million and rising.^1^ Patients with overt hypercortisolism present with fatigue, depression and anxiety, facial plethora, striae, obesity, hirsutism, psychiatric changes, and bone loss, as well as hypertension, hyperlipidemia, and diabetes mellitus that increase morbidity and mortality due to cardiovascular disease, thromboembolic events, and sepsis.^2^

Pharmacotherapy with steroidogenesis inhibitors such as metyrapone, ketoconazole, levoketoconazole, and osilodrostat to block adrenal synthesis of cortisol might be used when surgical tumor resection is not possible or not curable, as they reduce cortisol levels and improve signs and symptoms associated with hypercortisolism. However, careful monitoring is required to avoid adrenal insufficiency requiring treatment with hydrocortisone.^2^

Osilodrostat, a CYP11B1 inhibitor, is approved in the United States to treat Cushing’s disease caused by an ACTH-producing pituitary adenoma in patients for whom pituitary surgery is not curative or is not an option. In these patients, osilodrostat reduced cortisol levels in the first 2 weeks of treatment and normalized cortisol in 86% of patients by week 34 with improvement of hyperglycemia, hypertension, and hyperlipidemia by week 48. Hypocortisolism was demonstrated in 51% of patients treated with osilodrostat.^3^

Few publications have reported on the off-label use of osilodrostat in nonpituitary Cushing’s syndrome. Osilodrostat was shown to be highly effective in reducing cortisol levels in 8 patients with adrenal carcinoma, 3 of whom developed adrenal insufficiency.^4^ In a hospitalized patient with a malignant ACTH-secreting neuroendocrine tumor (NET), plasma cortisol was normalized within 6 days of initiating osilodrostat 15 mg twice daily. Hypocortisolism was seen 7 days after the dose was increased to 30 mg twice daily, but hydrocortisone 20 mg daily had been started on the fifth day of treatment to avoid symptoms. Cortisol decrease was accompanied by a decrease in insulin dose requirement.^5^

A phase 2, single-arm, open-label, dose titration study^6^ treated patients with Cushing’s syndrome due to adrenal adenoma (*n* = 5), ectopic ACTH-secreting NET (*n* = 3), and ACTH-independent macronodular adrenal hyperplasia (*n* = 1) with escalating doses of osilodrostat for up to 12 weeks, starting at 2 mg twice daily and increasing to 30 mg twice daily every week; dose could be reduced if cortisol levels were below the lower limit of normal. Overall, >80% reduction was seen in mean urinary free cortisol (mUFC) by week 4, with ACTH-independent macronodular adrenal hyperplasia and ACTH-secreting NET patients showing a 99% decrease and adrenal adenoma patients showing a 53%-98% decrease from baseline, although only modest reductions were seen in fasting blood glucose, HbA1c, and blood pressure. Adrenal insufficiency was the most common adverse event, including 2 patients (22%) with grade 3, and it was also the most common reason for dose reduction, treatment interruption, and initiation of glucocorticoids. Cortisol level below the lower limit of normal was detected in 3 patients, all of whom had adrenal adenoma. Other reported side effects of osilodrostat included hypokalemia, QT prolongation, hypertension, hyperandrogenism, and elevated liver transaminases.

Here, we present a patient with overt ACS treated with osilodrostat prior to adrenalectomy scheduled 3 weeks later in an attempt to reduce cortisol and severity of associated comorbidities, including poorly controlled diabetes mellitus and hypertension, and thereby decrease risk for perioperative morbidity and mortality.

## Case Report

A 48-year-old woman was admitted for vomiting and acute worsening of chronic right lumbar back pain. She reported a 6-year history of truncal weight gain requiring gastric bypass surgery and a 3-year history of diabetes mellitus, hypertension, hyperlipidemia, depression, and anxiety requiring metformin, liraglutide, amlodipine, indapamide, clonidine, potassium chloride, rosuvastatin, ezetimibe, aspirin, and lansoprazole, as well as tramadol as needed for back pain.

A 3.3 × 2.7 × 2.2 cm indeterminate right adrenal gland mass without the classic phase/out-of-phase dropout of a nonfunctioning adrenal adenoma had been found on abdomen magnetic resonance imaging 2 years earlier, and serum cortisol level of 17.3 μg/dL was seen after overnight dexamethasone suppression test performed 2 weeks prior to admission. Lower extremities arterial duplex showed diffuse atherosclerosis, and venous duplex showed venous reflux but not deep vein thrombosis.

On physical exam, blood pressure was 149/100 mmHg, pulse 85 bpm, weight 93.5 kg (206 lbs), height 152 cm (59 inches), and body mass index 40.5 kg/m^2^. Truncal obesity, moon facies with plethora and hirsutism, posterior cervicothoracic fat pad, thin fragile skin without purple striae, and alopecia were apparent. Laboratory tests were notable for white blood cell count 18.64 1000 U/L (normal, 4.0-11.0 1000 U/L), glucose 136 mg/dL (70-99 mg/dL), potassium 3.0 mmol/L (3.5-5.0 mmol/L), alkaline phosphatase 152 U/L (40-150 U/L), alanine transaminase 107 U/L (0-55 U/L), aspartate transaminase 41 U/L (5-34 U/L), and HbA1c 7.8% (<5.7%) (Table 1). Confirming the suspected diagnosis of ACS, repeat serum cortisol was 13.8 μg/dL after 1 mg oral dexamethasone suppression test (normal, ≤1.8 μg/dL), dexamethasone level 217 ng/dL (>200 ng/dL), urinary free cortisol 1324 μg/24 h (4.0-50.0 μg/24 h), ACTH <5 pg/mL (6-50 pg/mL), and dehydroepiandrosterone sulfate 10 μg/dL (19-231 μg/dL) (Table 2). Initial electrocardiography showed normal sinus rhythm with prolonged QT interval of 493 ms, which normalized with oral potassium repletion. Transthoracic echocardiography showed normal biventricular size and left ventricular ejection fraction without wall motion abnormalities but with possible mild right ventricular dysfunction. Abdominal computed tomography with contrast showed a 3.4 × 2.8 cm mildly heterogeneous indeterminate right adrenal gland mass.

**Table 1:** Laboratory Results on Admission

Test	Reference range	Result
White blood cells, 1000 U/L	4.0-11.0	18.64
Neutrophils, 1000 U/L	1.80-8.00	12.26
Hemoglobin, g/dL	11.6-15.4	14.4
Platelets, 1000 U/L	150-450	444
Glucose, mg/dL	70-99	136
Sodium, mmol/L	135-145	139
Potassium, mmol/L	3.5-5.0	3.0
Creatinine, mg/dL	0.57-1.11	0.60
Magnesium, m/dL	1.6-2.6	1.8
Alkaline phosphatase, U/L	40-150	152
Alanine transaminase, U/L	0-55	107
Aspartate transaminase, U/L	5-34	41
HbA1c, %	<5.7%	7.8

**Table 2:** Laboratory Results Supporting a Diagnosis of Adrenal Cushing’s Syndrome

Test	Reference range	Result
24-h urinary free cortisol, μg/24 h	4.0-50.0	1324
0800 cortisol level after 1 mg DST, μg/dL^a^	≤1.8	13.8
Dexamethasone level after 1 mg DST, ng/dL	≤20	217
Adrenocorticotropic hormone, pg/mL	6-50	<5
Dehydroepiandrosterone sulfate, μg/dL	19-231	10
Total testosterone, ng/dL	2-45	3
Free testosterone, pg/mL	0.1-6.4	0.7
Aldosterone, ng/dL	0.0-30.0	5.8
Plasma renin activity, ng/mL/h	0.167-5.380	1.118
Aldosterone/renin ratio, ng/dL per ng/mL/h	0.0-30.0	5.2
Fractionated free plasma normetanephrines, pg/mL	0.0-125.8	30
Fractionated free plasma metanephrines, pg/mL	0.0-88.0	<10

DST = dexamethasone suppression test.

24 hour urine creatinine 3.41 g/24 h (0.5-2.15), total urine volume 1735 mL.

a 0800 dexamethasone level, 217 ng/dL (>200 ng/dL validates test results).

To reduce severity of comorbidities associated with cortisol-induced poorly controlled diabetes mellitus and hypertension prior to adrenalectomy and thereby reduce risk for perioperative morbidity and mortality, preoperative treatment with a steroidogenesis inhibitor was considered. Ketoconazole was avoided due to her increased liver transaminases, and metyrapone was avoided due to the potential difficulty in swallowing multiple large tablets in the setting of nausea and concern for her lack of compliance. We therefore selected osilodrostat, which is given as small tablets and is dosed starting at 2 mg twice daily, then titrated by 1-2 mg twice daily every 2 weeks up to a maximum of 30 mg twice daily.

Osilodrostat 2 mg twice daily was initiated on the day of hospital discharge (treatment day 1). Sulfamethoxazole/trimethoprim 800 mg/160 mg daily was added for pneumocystis pneumonia prophylaxis, and glimepiride was added for additional glycemic control. The patient was instructed to record daily heart rate, blood pressure, and blood glucose levels at home.

At a follow-up appointment on treatment day 6, the patient denied any change in symptoms or side effects. Morning cortisol was 13.9 μg/dL, and the osilodrostat dose was increased to 4 mg twice daily. On treatment day 9, she reported severe fatigue, nausea and vomiting, poor appetite, exertional palpitations, shortness of breath, and weight loss, and morning cortisol was 7.2 μg/dL, all of which were suspicious for adrenal insufficiency. She was treated with intravenous hydrocortisone 50 mg and saline and these symptoms resolved. She was instructed to decrease osilodrostat from 4 to 2 mg twice daily and to add oral hydrocortisone 15 mg in the morning and 5 mg in the afternoon to her treatment regimen, which continued until adrenalectomy.

Figure depicts the decline in morning cortisol and fasting glucose levels over the 22 days of treatment with osilodrostat. Morning cortisol declined from 19.2 μg/dL on treatment day 1 to 5.1 μg/dL on treatment day 22, 1 day prior to adrenalectomy. (To avoid cross-reactivity, cortisol was measured at 0800, which was 16 hours after the last hydrocortisone dose.) She was unable to properly collect urine for 24 hours, so mUFC was not measured. ACTH remained suppressed. Fasting glucose decreased from 224 mg/dL prior to starting osilodrostat (treatment day 1) to 122 mg/dL prior to adrenalectomy (treatment day 23), and all antidiabetic medications were self-discontinued by treatment day 19. She reported no significant changes in home blood pressure measurements, and antihypertensive medication regimen during osilodrostat treatment remained unchanged.

**Fig:**
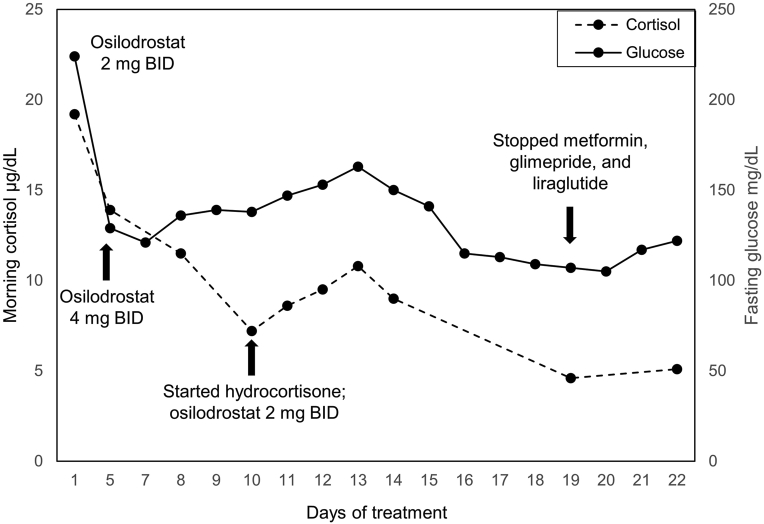
Morning serum cortisol and fasting glucose levels during osilodrostat therapy (treatment day 1 through day 22). Morning cortisol was measured after at least 16 h from the last hydrocortisone dose and after overnight fasting. *BID* = twice daily.

Osilodrostat was discontinued on the day prior to adrenalectomy (treatment day 23). The patient received 100 mg intravenous hydrocortisone during surgery followed by hydrocortisone dose taper over the next 6 days to achieve a maintenance dose of daily hydrocortisone 20 mg. She did not develop any perioperative complications and was discharged with instructions to take enoxaparin for 14 days for thromboprophylaxis. Surgical pathology confirmed a 3.4 cm focally pigmented adrenocortical adenoma.

At follow-up 11 months after surgery, the patient had lost 9.1 kg (23 lbs), HbA1c decreased to 5.8%, and she did not require any antidiabetic medications. Blood pressure improved to 106/66 mmHg on amlodipine 2.5 mg daily. Morning cortisol 16 hours after the last hydrocortisone dose was 0.1 μg/dL, yet, for the first time, ACTH level was detectable at 4.3 pg/mL (7.2-63.3 pg/mL), indicating partial recovery of the hypothalamus-pituitary-adrenal axis. Maintenance dose of hydrocortisone will continue until adrenal function recovery.

## Discussion

We describe a 48-year-old woman with ACTH-independent Cushing’s syndrome due to an adrenal adenoma who was treated with off-label osilodrostat to reduce cortisol levels and severity of poorly controlled diabetes mellitus and hypertension prior to adrenalectomy. Other available off-label steroidogenesis inhibitors, including ketoconazole and metyrapone, were deemed less appropriate given her clinical state. Levoketoconazole was not yet approved at the time but has hepatic-related concerns similar to ketoconazole, making it a less attractive choice for our patient with elevated transaminases. During the 3 weeks prior to adrenalectomy, the patient adhered to the osilodrostat and then hydrocortisone treatment regimen, as well as to the glucose and blood pressure self-monitoring regimen, although she was not able to properly collect urine for 24 hours. By the day of surgery, morning cortisol levels had markedly decreased, and fasting glucose levels improved sufficiently to stop antidiabetic medications, potentially allowing for more favorable perioperative outcomes, even though hypertension did not improve.

Our patient developed a clinical presentation suggestive of adrenal insufficiency, which is a recognized possible adverse effect of osilodrostat. The presentation could have also suggested glucocorticoid withdrawal, a state of relative hypocortisolism that might be seen in patients with long-term Cushing’s syndrome who undergo surgery to remove a tumor causing hypercortisolism.^7^ Nevertheless, both conditions can be safely treated with hydrocortisone replacement. In retrospect, it appears that osilodrostat 2 mg twice daily plus hydrocortisone (in a block-and-replace strategy^8^) might have been sufficient, and osilodrostat dose escalation might have not been necessary, despite the patient’s very high 24-hour mUFC at presentation.

Of note, our patient did not manifest other adverse effects associated with osilodrostat use, including hypokalemia, hypertension, and hirsutism from the accumulation of androgen precursors. This might be due to the relatively short duration of 3 weeks of therapy and/or due to the low dose used.

There is no clear guidance or published evidence describing potential benefits of steroidogenesis inhibitor therapy to reduce ACS severity and risk for perioperative complications before adrenalectomy. Nevertheless, consensus guidelines on management of Cushing’s syndrome supports the use of preoperative treatment if surgery is delayed or if the patient manifests a potentially life-threatening morbidity such as metabolic, infectious, cardiovascular, thromboembolic, or psychiatric complications.^2^ In addition, improved perioperative glycemic control has been shown to contribute to reduced length of hospital stay and reduced risk of postoperative infection, stroke, and mortality in general.^9^^,^^10^ Thus, our use of preoperative osilodrostat in this patient with overt ACS might have reduced some risk of perioperative complications.

## Conclusion

In patients with an ACTH-independent, cortisol-producing adrenal adenoma awaiting adrenalectomy, osilodrostat might be considered to rapidly control cortisol and disease-associated hyperglycemia. Close monitoring for signs and symptoms of adrenal insufficiency or glucocorticoid withdrawal is important, even with low treatment doses, and the proactive addition of hydrocortisone should be considered. As cortisol-secreting adrenal adenomas are not uncommon, further studies are warranted to evaluate osilodrostat effect on surgery-related morbidities and outcomes.

## Disclosure

A.B. has served as a member of an advisory board for Recordati.
